# NIR-triggered gelatin-based hydrogel for ion-mechanical synergistic antibacterial therapy and infected wound closure

**DOI:** 10.1039/d6ra07136h

**Published:** 2026-10-19

**Authors:** Xiaoxiao Hu, Lemin Zhang, Ruixin Hao, Zeyu Wang, Qiuhong Zhang, Zhicheng Gao, Wei Yang, Yongping Ma

**Affiliations:** a Department of Clinical Pharmacy, The No.2 Hospital of Baoding Baoding 071052 People's Republic of China; b School of Mechanical Engineering, Dalian University of Technology Dalian 116024 China; c School of Physics and Materials, Nanchang University Nanchang 330031 China; d College of Stomatology, North China University of Science and Technology Tangshan 063200 People's Republic of China 3201165077@qq.com; e Respiratory Medicine, The No.2 Hospital of Baoding Baoding 071052 People's Republic of China hdfyyw@sina.com; f Department of Stomatology, The No.2 Hospital of Baoding Baoding 071052 People's Republic of China

## Abstract

Bacterial infection and impaired wound closure remain the leading causes of refractory cutaneous wound healing disorders, inflicting substantial global morbidity and mortality while exacerbating the crisis of antibacterial resistance. Infected cutaneous wounds present a persistent clinical difficulty, given that bacterial infection and inadequate wound closure exacerbate one another. Here, we constructed a hydrogel platform that integrates antibacterial activity and active wound closure through ion-mechanical coupling. The system combines thermoresponsive, catechol-functionalized network with core–shell nanoparticles consisting of a metal–organic framework shell (MOF) encapsulating a liquid metal core (LM). Under near-infrared (NIR) irradiation, the liquid metal generates heat that triggers network contraction to mechanically close the wound. Simultaneously, sustained release of Zn^2+^ and Ga^3+^ from the nanoparticles provides continuous, antibiotic free antibacterial activity. In a mouse MRSA infected wound model, this hydrogel achieves complete closure within 14 days with improved tissue regeneration. By integrating ion mediated antibacterial action, photothermal bacterial inactivation, and active mechanical contraction in a single platform, this design addresses the intertwined challenges of infection and impaired closure without the compromises of conventional approaches.

## Abstract 2

In this work, we constructed a NIR-triggered MOF/liquid metal hydrogel platform integrating photothermal mechanical contraction with Zn^2+^/Ga^3+^ release for synergistic antibacterial therapy and infected wound repair.

## Graphical abstract

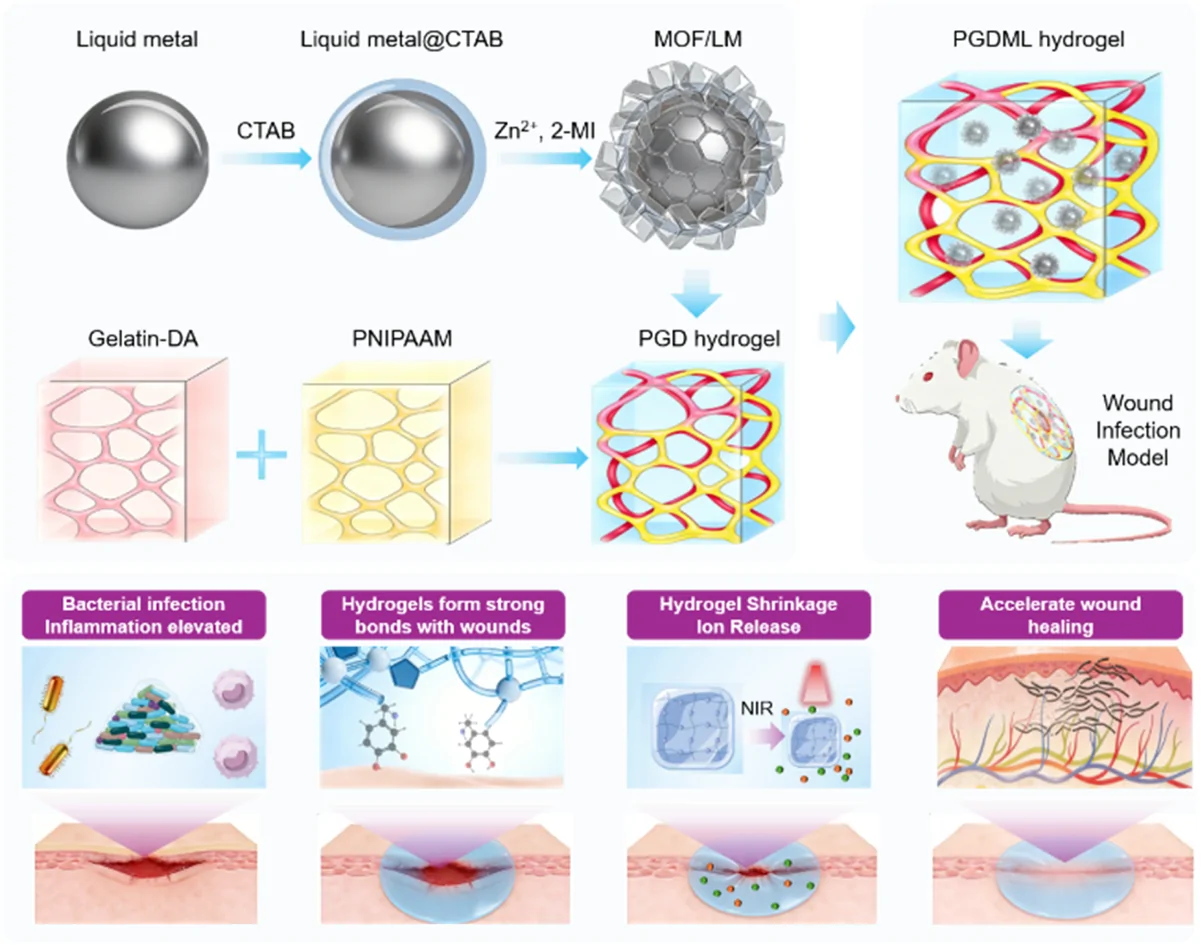

## 1. Introduction

Bacterial infections of cutaneous wounds, particularly those caused by drug-resistant pathogens and biofilm formation, represent a growing global clinical burden, with chronic non-healing wounds (including diabetic foot ulcers, pressure injuries, and surgical site infections) being a leading cause of amputation, sepsis, and mortality worldwide.^1,2^ The World Health Organization (WHO) has formally warned that the unregulated global dissemination of antibacterial resistance (AMR) risks precipitating a post-antibiotic era, in which even uncomplicated cutaneous and soft-tissue infections will no longer be amenable to treatment with standard-of-care antibiotic regimens.^3,4^ Infected cutaneous wounds are defined by a vicious cycle: bacterial colonization delays tissue repair, and delayed closure leaves the wound vulnerable to persistent infection.^5^ Standard treatment addresses these two problems separately-antibiotics target pathogens, while passive dressings maintain a moist environment-but neither intervention actively closes the wound.^6,7^ Addressing that could break this cycle by simultaneously eliminating infection and promoting active wound closure would address a critical unmet need.

Hydrogel-based wound dressings have emerged as a promising platform for infected wound management, owing to their intrinsic ability to maintain a moist wound microenvironment, absorb exudate, and serve as a depot for localized delivery of therapeutic agents.^8–15^ To date, numerous hydrogel systems have been developed incorporating antibacterial modalities including photothermal therapy (PTT),^16–18^ metal ion-based antibacterial agents,^19,20^ and antibacterial peptides.^21–23^ However, single-modality antibacterial approaches often require high doses or prolonged treatment to achieve sufficient efficacy, increasing the risk of off-target damage (*e.g.*, non-specific thermal injury to healthy tissue in PTT) and acquired resistance.^24^ Besides, existing hydrogel designs typically treat antibacterial activity and wound contraction as independent processes, failing to exploit the mechanical and chemical synergies that could amplify therapeutic efficacy while minimizing side effects.^25,26^ In particular, the potential to couple thermoresponsive hydrogel contraction with on-demand antibacterial ion release-an ion force coupling mechanism-remains largely unexplored in the field.^27–29^

Owing to their superior photothermal conversion capability, gallium based liquid metal nanoparticles (LMNPs) represent promising candidates for infected wound nanomedicine, delivering photothermal effects and bactericidal Ga^3+^ release. Nevertheless, bare LMNPs suffer from irreversible aggregation and uncontrolled leakage under physiological aqueous conditions, which deteriorate composite stability upon hydrogel integration. Moreover, bare LMNPs only supply gallium ions, lacking supplementary antibacterial metal sources for enhanced anti-infective performance.^30^ A viable strategy to mitigate the inherent drawbacks mentioned above is to construct composite nanostructures by combining liquid metal nanoparticles with other functional components. To date, extensive research has confirmed that the actual performance of liquid metal nanoparticles depends heavily on their surface functionalization. Consequently, much previous work has employed core–shell structure engineering to overcome the instability caused by the fragile oxide layer on the surface of pristine liquid metals.

As a representative subclass of metal organic frameworks, zeolitic imidazolate framework 8 (ZIF 8) has gained tremendous attention in antibacterial biomaterial research owing to its intrinsic structural merits, including high porosity, tunable pore architecture and favourable biosafety. Retaining structural integrity under neutral physiological conditions, ZIF-8 undergoes progressive framework dissociation within acidic microenvironments typical of bacterial infected wounds, enabling pH triggered liberation of bactericidal Zn^2+^. Such infection responsive ion output endows ZIF-8 with inherent antimicrobial potency without relying on conventional antibiotic agents.^31^ Benefiting from its porous crystalline lattice, ZIF-8 is widely exploited as a protective encapsulating shell for diverse inorganic nanocores.

Herein, we constructed core–shell nanoparticles consisting of a metal–organic framework shell (MOF, ZIF-8) encapsulating a liquid metal core (LM), embedded within a thermoresponsive, catechol-modified gelatin and poly(*N*-isopropylacrylamide) network (PNIPAAm-Gel-DA). Under near-infrared (NIR) irradiation, the liquid metal core heats rapidly, triggering localized contraction of the thermoresponsive network. This contraction is transmitted directly to the wound edges *via* the adhesive gelatin component, delivering active mechanical force to close the defect. Meanwhile, the ZIF-8 shell and liquid metal core release Zn^2+^ and Ga^3+^ ions, providing sustained antibacterial activity against drug-resistant pathogens without the use of conventional antibiotics (Fig. 1). In addition, the photothermal effect generated under NIR irradiation also contributes directly to bacterial inactivation, further enhancing the overall antibacterial capacity of the platform. We evaluate this system in a mouse model of full-thickness wounds infected with methicillin-resistant *Staphylococcus aureus* (MRSA). This hydrogel exhibits excellent antibacterial properties, enabling complete wound healing within 14 days while accelerating epithelialization and promoting collagen deposition. By separating the activation of mechanical contraction from the sustained release of antibacterial ions, this design offers a simple strategy for the repair of infected wounds that addresses both infection and healing barriers.

**Fig. 1:**
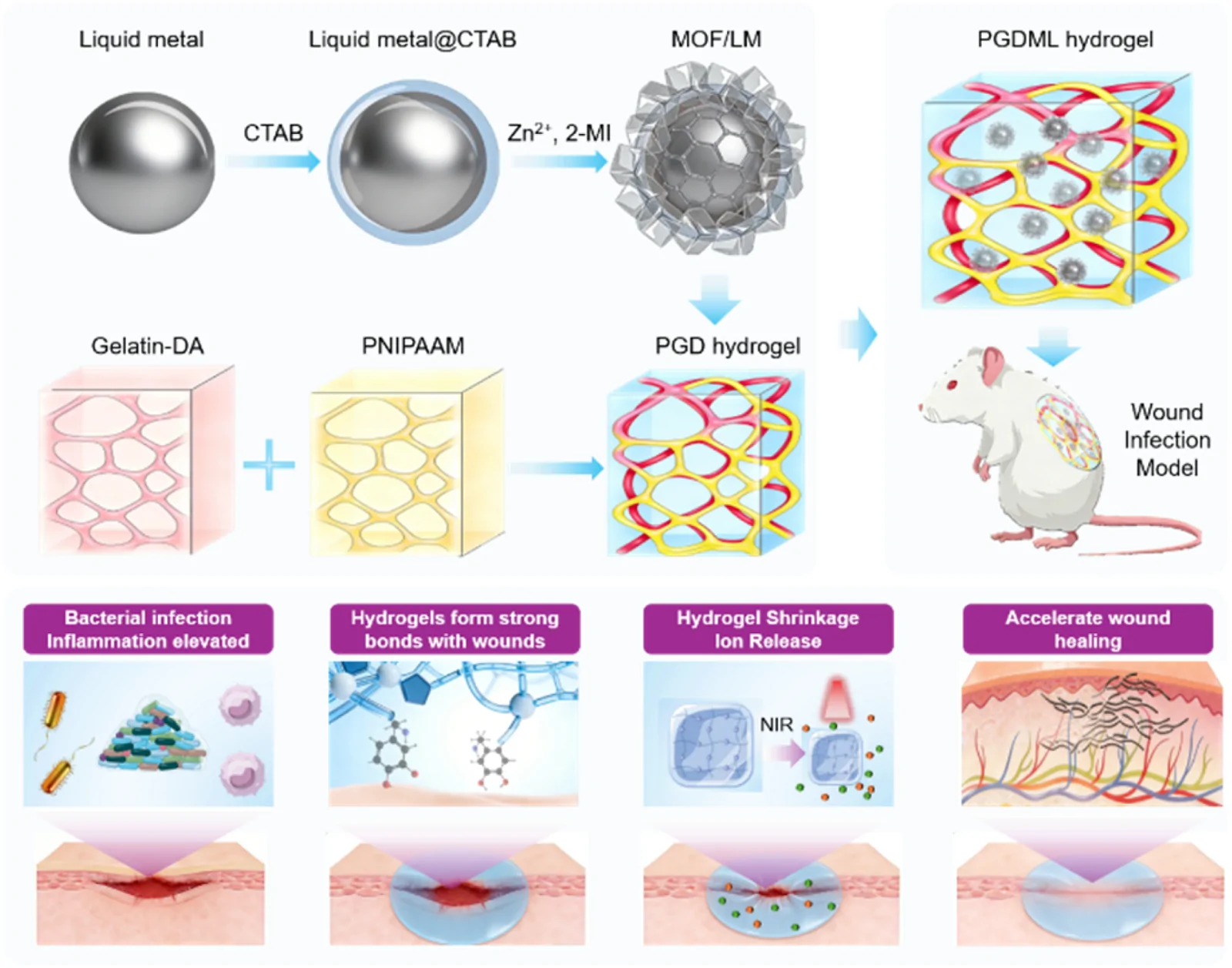
Schematic diagram of the preparation process for MOF/LM based hydrogels and their mechanisms action in reducing inflammation and promoting healing in infected wounds.

## 2. Materials and methods

### 2.1 Materials

Eutectic gallium indium (EGaIn) and zinc nitrate hexahydrate (Zn(NO_3_)_2_·6H_2_O) were obtained from Shanghai Maclin. 2-Methylimidazole (2-MI), Gelatin (Gel), dopamine hydrochloride (DA), *N*-isopropyl acrylamide (PNIPAAM), *N*-(3-dimethylaminopropyl)-*N*′-ethylcarbodiimide hydrochloride (EDC), *N*-hydroxysuccinimide (NHS) and methanol were bought from Aladdin. Ammonium persulfate (APS) and *N*,*N*,*N*′,*N*′-tetramethylethylenediamine (TEMED) were purchased from Sigma-Aldrich, L929 mouse fibroblasts and the culture medium kit were obtained from Wuhan Servicebio Technology Co., Ltd (Wuhan, China).

### 2.2 Synthesis of core–shell MOF/liquid metal nanoparticles (MLNPs)

MLNPs was synthesized *via* a two-step protocol. Briefly, 20 µL of EGaIn was added to 2 mL of ultrapure water pre-dissolved with 3.6 mg of CTAB. The mixture was then treated with a digital ultrasonic homogenizer (SCIENTZ-IID, Ningbo Scientz Biotechnology) at 40% amplitude for 30 min under a pulse mode (3 s on and 6 s off) to obtain CTAB-stabilized LM nanoparticles. For ZIF-8 coating, two stock solutions were prepared separately by dissolving 2.168 g of 2-MI and 0.146 g of Zn(NO_3_)_2_·6H_2_O in 20 mL of ultrapure water, respectively. The Zn(NO_3_)_2_·6H_2_O stock solution was rapidly poured into the 2-MI solution, followed by 15 s of vigorous vortex homogenization to form ZIF-8 seed suspension. The as-prepared EGaIn nanoparticles dispersion was subsequently added to the suspension, with an additional 15 s of vortex mixing to ensure full homogenization. The reaction mixture was incubated undisturbed at room temperature for a minimum of 10 h to complete the encapsulation process. Finally, MLNPs were collected by centrifugation at 5000 rpm for 10 min and washed with ultrapure water.^32^

### 2.3 Synthesis of dopamine-modified gelatin (Gel-DA)

Gel-DA was synthesized *via* carbodiimide-mediated amidation to graft DA moieties onto gelatin backbones. Briefly, 4 g gelatin was first dissolved in 100 mL PBS buffer (pH 7.4) at 50 °C. Then, 1 g of EDC and 0.6 g NHS were added, followed by 30 min activation under magnetic stirring. After, 2 g of dopamine hydrochloride was then introduced, and the reaction proceeded for 24 h at 25 °C, the solution maintains the pH at 5.0–6.0. The product was dialyzed against ultrapure water for 72 h with dialysate refreshed every 6 h to remove unreacted reagents. The final Gel-DA was obtained *via* lyophilization.

### 2.4 Fabrication of MOF/LM incorporated adhesive thermoresponsive hydrogels

The PNIPAAM-Gel-DA-MOF/LM (PGDML) composite hydrogel was fabricated *via* one-step *in situ* free radical polymerization, with ammonium persulfate (APS) as the initiator and *N*,*N*′-methylenebisacrylamide (MBA) as the crosslinker. Briefly, 1.2 g of *N*-isopropylacrylamide (NIPAAm) monomer, 12 mg of MBA crosslinker, and 0.6 g of the as-prepared Gel-DA conjugate were sequentially dissolved in 10 mL of ultrapure water under stirring at room temperature. The as-synthesized ZIF-8 encapsulated liquid metal core–shell nanoparticles were then added to the mixture, followed by 10 min of ultrasonic dispersion in an ice bath to obtain a homogeneous suspension. After that, 24 mg of APS initiator was added to the deoxygenated mixture and mixed rapidly and evenly. The pre-gel solution was immediately injected into a custom polytetrafluoroethylene mold, and incubated at 4 °C for 6 h to complete free radical polymerization and form the composite hydrogel. The pure PNIPAAM-Gel-DA (PGD) hydrogel without MOF/LM nanoparticles were prepared as the control group following the same procedure.

### 2.5 Characterization of core–shell MOF/LM nanoparticles

The morphologies of MOF/LM nanoparticles and freeze-fractured cross-sections of the hydrogels were characterized *via* field-emission scanning electron microscopy (FE-SEM, SU8600). Transmission electron microscopy (TEM, JEM F200) coupled with energy-dispersive X-ray spectroscopy (EDX) were performed to characterize the morphology, core–shell nanostructure, and elemental distribution of LM and MOF/LM nanoparticles. Zeta potential was measured *via* dynamic light scattering (DLS, Zetasizer Nano ZS 90).

### 2.6 Characterization of PGDML hydrogels

The porous microstructure and LMZ dispersion in hydrogels were observed by cryogenic SEM (SU5000). Tensile and compressive mechanical properties were tested using a universal material testing machine (DR-502AD) with standard specimens. Dynamic rheological properties were measured *via* a rotational rheometer (Anton Paar MCR 302) with strain, frequency, and temperature sweeps to characterize viscoelasticity. Wet tissue adhesive strength was quantified by lap-shear and 90° peeling tests on fresh porcine skin, which was purchased from a local market as a by-product of food production; no animals were killed specifically for this study. Photothermal performance was evaluated under 808 nm NIR laser irradiation (1.0 W cm^−2^, 5 min), with real-time temperature recorded by an infrared thermal camera (FLIR T640), all photothermal experiments were performed under hydrated conditions the hydrogels were equilibrated in PBS (pH 7.4) at 25 °C for 24 h prior to NIR irradiation. The equilibrated swelling ratio (ESR) of the hydrogels was determined by swelling tests. The freshly prepared hydrogels (200 µL, *n* = 5) were immersed in PBS (pH 7.4) at 37 °C. At pre-determined times, the samples were taken out and the swollen weights were recorded after removing the excessive superficial water with a filter paper. Zn^2+^ and Ga^3+^ release content in PBS (pH 7.4 and 5.5) were quantified *via* UV-Vis spectrophotometer (Cary 100).

### 2.7 *In vitro* antibacterial and anti-biofilm assays

Gram-negative *Escherichia coli* (*E. coli*, ATCC 25922) and Gram-positive *Staphylococcus aureus* (*S. aureus*, ATCC 25923) were used in this study. All bacterial strains were cultured in sterile Luria-Bertani (LB) broth at 37 °C with constant shaking at 220 rpm. Bacteria in the mid-logarithmic growth phase were collected, washed twice with sterile PBS (pH 7.4), and adjusted to the required concentration for subsequent experiments. The bactericidal performance of the hydrogels was quantified *via* the standard colony-forming unit (CFU) counting method. 4 groups were set for the assay: (1) PBS; (2) PGD hydrogel; (3) PGDML composite hydrogel; (4) PGDML with NIR irradiation (PGDML + NIR).

Prior to the assay, hydrogel discs (10 mm in diameter, 1 mm in thickness) were sterilized *via* 1 h of UV irradiation on each side. Each sample was incubated with 1 mL of bacterial suspension (1 × 10^6^ CFU mL^−1^ in LB broth) in a 24-well plate at 37 °C for 4 h. For NIR-treated groups, samples were irradiated with an 808 nm fiber-coupled laser at 1.0 W cm^−2^ for 5 min after 1 h of pre-incubation. After incubation, the bacterial suspension was serially diluted 10-fold with sterile PBS, and 100 µL of the appropriate dilution was spread on LB agar plates. After 18 h of incubation at 37 °C, the number of visible colonies was counted, and the bactericidal rate was calculated using the following formula:Antibacterial rate (%) = (*N*_control_ − *N*_sample_)/*N*_control_ × 100%where *N*_control_ is the CFU count of the PBS group, and *N*_sample_ is the CFU count of the experimental group.

### 2.8 Biofilm clearance assay

The anti-biofilm performance of the hydrogels was evaluated against 24 h pre-formed mature biofilms by using crystal violet (CV) staining. Briefly, 200 µL of bacterial suspension (1 × 10^7^ CFU mL^−1^) was added to a 96-well polystyrene plate and incubated at 37 °C for 24 h to allow the formation of mature, intact biofilms. The planktonic bacteria were then removed, and the wells were gently washed 3 times with sterile PBS to remove non-adherent bacteria. Sterile hydrogel discs (6 mm in diameter) and 200 µL of fresh LB broth were added to each well, with the same grouping as the planktonic bactericidal assay. After 24 h of incubation at 37 °C (with NIR irradiation administered as described above), the supernatant was removed, and the wells were washed 3 times with PBS.^33^

For semi-quantitative analysis of biofilm biomass: biofilms were fixed with 4% paraformaldehyde for 30 min, stained with 0.1% (w/v) CV solution for 15 min at room temperature, and washed thoroughly with PBS to remove unbound dye. The bound CV was dissolved in glacial acetic acid, and the absorbance at 570 nm (OD_570_) was measured using a microplate reader (Tecan Infinite M200 Pro). The biofilm clearance rate was calculated as:Biofilm clearance rate (%) = (OD_control_ − OD_sample_)/OD_control_ × 100%

### 2.9 *In vivo* bacterial-infected wound healing evaluation

All animal procedures were performed in accordance with the Guidelines for Care and Use of Laboratory Animals of Dalian Municipal Central Hospital and approved by the Animal Ethics Committee of Dalian Municipal Central Hospital. Female Kunming mice weighing 35–40 g and 5–6 weeks age were used for assay. All mice were housed in a temperature-controlled environment (23 ± 2 °C, 50 ± 5% humidity) with a 12 h light/dark cycle, with free access to sterile food and water. Mice were anesthetized by intraperitoneal injection of 1% sodium pentobarbital (50 mg kg^−1^ body weight). The dorsal hair was removed and the skin was disinfected with 75% ethanol and povidone–iodine. A full-thickness circular skin defect (including the epidermis, dermis, and panniculus carnosus) was created on the dorsal midline of each mouse using a sterile biopsy punch. 50 µL of *S. aureus* suspension (1 × 10^8^ CFU mL^−1^) was inoculated onto the wound area to establish a stable, clinically relevant *S. aureus*-infected wound model.

Mice were randomly divided into 4 groups (*n* = 6 per group) using a random number table: blank control group, PGD group, PGDML group, PGDML + NIR group. Hydrogel discs (8 mm in diameter, 1 mm in thickness) were applied to the wound area. Treatments were administered once daily for 14 consecutive days. Treatment consisted of 100 µL injections administered *via* the tail vein every other day.

The *in vivo* photothermal conversion performance of the hydrogels was monitored during the first treatment. Real-time temperature changes at the wound site were recorded during 5 min of NIR irradiation using an infrared thermal camera (FLIR T640, FLIR Systems). Wound images were captured with a digital camera on day 0 (model establishment), 5, 10, and 14 after treatment. On day 5 post-treatment, 3 mice from each group were euthanized. The wound tissues were harvested, weighed, homogenized in 1 mL of sterile PBS, and serially diluted for CFU counting on LB agar plates, as described above. On day 14 post-treatment, all remaining mice were euthanized. The full-thickness wound tissues, including the surrounding normal skin, were harvested, fixed in 4% paraformaldehyde for 24 h, dehydrated in gradient ethanol, cleared with xylene, and embedded in paraffin. Serial sections (5 µm thick) were prepared for histological staining.

### 2.10 Statistical analysis

All quantitative data were expressed as mean ± standard deviation (SD). Statistical significance was defined as *p < 0.05, **p < 0.01, ***p < 0.001. All experiments were performed with at least 3 independent biological replicates to ensure the reliability of the results.

## 3. Result and discussion

### 3.1 Physicochemical characterization of functional nanoparticles and composite hydrogel

The morphology of the pristine ZIF-8 nanoparticles was characterized by FE-SEM. As shown in the high-magnification SEM image, the prepared ZIF-8 nanoparticles exhibited a highly uniform regular rhombic dodecahedron morphology (the typical topological structure of ZIF-8 crystals) (Fig. 2a). LMNPs presented a spherical morphology with good dispersity (Fig. 2b). After the *in situ* growth of MOF on the LM surface, the resulting MOF/LM nanocomposites retained the core–shell structure, where the LM core was uniformly encapsulated by a rough, flower-like MOF shell (Fig. 2c), confirming the successful coating of MOF on LM. X-ray photoelectron spectroscopy (XPS) was employed to analyze the elemental composition and chemical states of the MOF/LM nanocomposites (Fig. 2d). The survey spectrum clearly showed the characteristic peaks of Zn 2p (from MOF) and Ga 2p/In 3d (from LM), demonstrating the coexistence of MOF and LM components in the nanocomposite (Fig. 2d). High-resolution XPS spectra of the MOF/LM nanocomposites reveal characteristic spin–orbit splitting peaks of In 3d and Ga 2p from the metallic LM core, as well as typical Zn 2p peaks from the MOF shell, verifying the successful integration of both functional components in the core–shell nanostructure (Fig. S1).

**Fig. 2:**
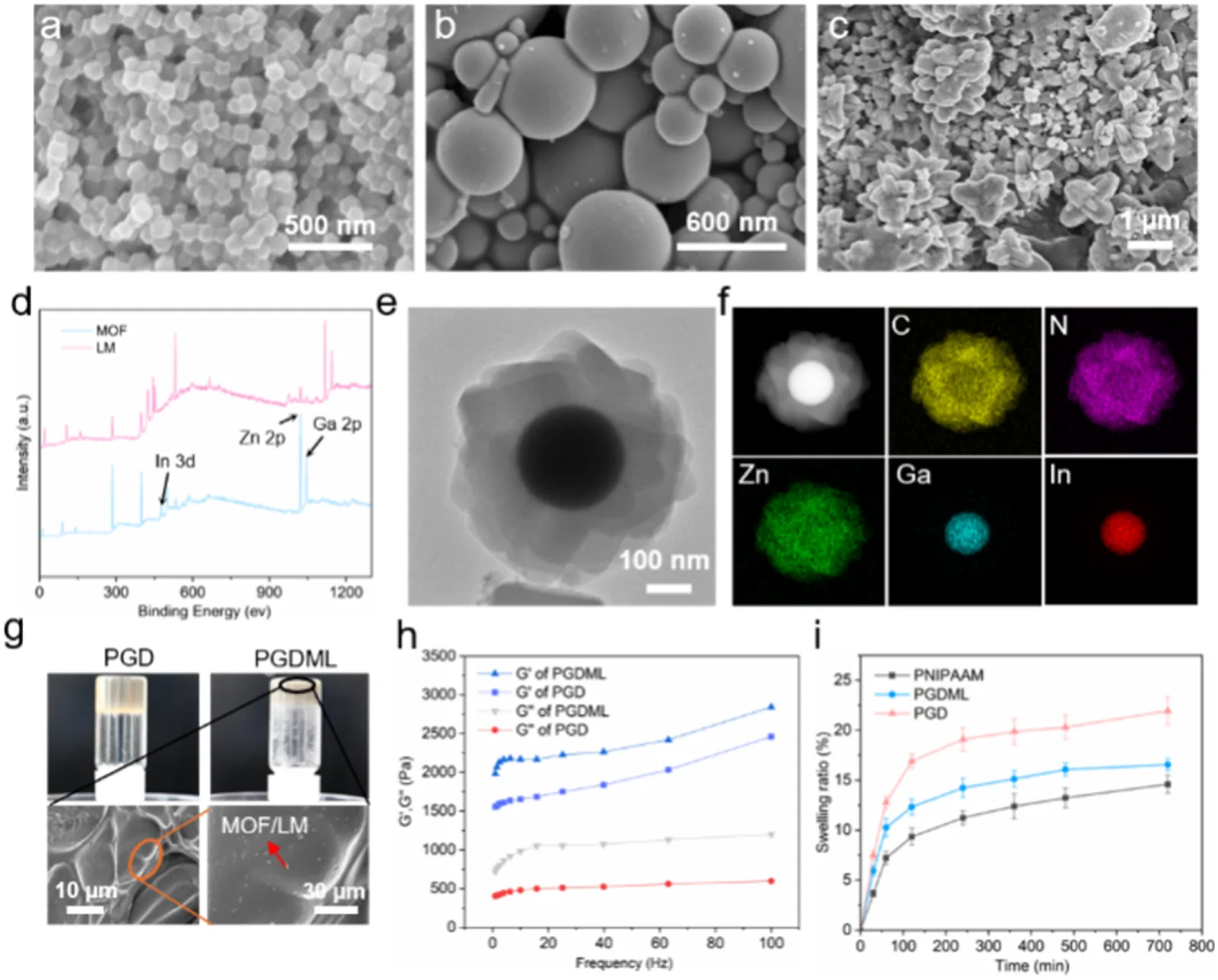
(a–c) SEM images of MOF, LM@CTAB and MOF/LM. (d) XPS spectrum of MOF/LM. (e) HADDF-STEM images and (f) corresponding elemental mappings of MOF/LM. (g) SEM images of PGDML hydrogel. (h) *G*′ and *G*″ of different hydrogels with frequency sweep. (i) Swelling ratio of different hydrogels within 720 min (*n* = 3).

The internal fine structure of a single MOF/LM nanoparticle was further characterized by TEM. The TEM image clearly revealed a well-defined core–shell hierarchical structure: the inner region was an electron-dense, high-contrast solid core. This unique core–shell structure perfectly combines the functional characteristics of the two components: the liquid metal core endows the nanoparticles with excellent photothermal conversion performance, free radical scavenging ability and mechanical flexibility, while the porous ZIF-8 shell not only provides physical protection for the liquid metal core to avoid its leakage and aggregation in the physiological environment, but also offers a high specific surface area and abundant active sites for antibacterial Zn^2+^ sustained release (Fig. 2e). EDX elemental mapping was performed to verify the elemental composition and spatial distribution of the MOF/LM nanoparticles. The mapping results showed that core constituent elements were detected in the nanoparticles, including Zn (from the metal node of the ZIF-8 framework), Ga and In (from the liquid metal core), as well as C, N (from the 2-methylimidazole organic ligand of ZIF-8). Spatially, Ga and In elements were mainly concentrated in the central core region of the nanoparticle, while Zn, C and N elements were uniformly distributed in the outer shell region, and all elements showed no obvious segregation, local aggregation or phase separation throughout the nanoparticle (Fig. 2f). This element distribution pattern is highly consistent with the core–shell structure observed in TEM. Zeta potential measurements revealed that pristine LM nanoparticles possessed a positive surface potential of ∼26 mV, which was markedly elevated to ∼39.5 mV after CTAB functionalization, verifying the successful modification of cationic surfactant on the LM surface that provides favorable electrostatic sites for the subsequent *in situ* growth of MOF; the final core–shell MOF/LM nanocomposites exhibited a surface potential of ∼25.5 mV, which lies between that of pure MOF nanoparticles (∼24.3 mV) and LM@CTAB, further confirming the successful construction of the MOF-coated LM core–shell nanostructure (Fig. S2).

We further prepared the MOF/LM doped PGD hydrogel (PGDML). The microstructure of the freeze-dried hydrogels was characterized by SEM. The results showed that the PGDML composite hydrogel exhibited a typical three-dimensional (3D) interconnected hierarchical porous structure with continuous and intact pore walls (Fig. 2g), which fully met the structural requirements of ideal wound dressing materials,^34^ and uniformly dispersed nanoparticles were observed to be embedded within the hydrogel network. The frequency sweep rheological test was performed in the range of 0.1–100 Hz. The storage modulus (*G*′) of both PGD and PGDML hydrogels was consistently higher than the corresponding loss modulus (*G*″) over the entire frequency range, indicating a typical elastic gel-like behavior and stable 3D crosslinked network. PGDML hydrogel exhibited significantly higher *G*′ and *G*″ values than PGD hydrogel,^35^ and the modulus of both hydrogels increased slightly with rising frequency, demonstrating excellent structural stability in a wide frequency range (Fig. 2h). The swelling behavior hydrogels was characterized in aqueous media. All hydrogels displayed rapid initial water uptake within the first 100 min, followed by a slowed hydration rate until reaching swelling equilibrium. The PGD hydrogel exhibited the highest equilibrium swelling ratio. The markedly elevated swelling capacity of PGD relative to neat PNIPAAm is attributed to the incorporation of dopamine-grafted gelatin, which introduces abundant hydrophilic moieties (phenolic hydroxyls, amino groups, and carboxyls) into the PNIPAAm network, substantially enhancing water binding and retention. The reduced equilibrium swelling of PGDML compared with PGD arises from MOF/LM nanoparticle incorporation: these nanoparticles act as additional physical crosslinking nodes *via* hydrogen bond and hydrophobic interactions with the polymer matrix.^36,37^ This increased crosslinking density restricts the relaxation and expansion of hydrogel chains in aqueous media, offsetting the hydrophilic contribution of the nanoparticles to yield a swelling ratio that remains significantly higher than that of neat PNIPAAm (Fig. 2i).

### 3.2 Photothermal properties of the composite hydrogel

The NIR-responsive photothermal performance and light-induced contraction behavior of the hydrogels were systematically characterized. Schematic illustration (Fig. 3a) depicts the light-triggered contraction process of the PGDML hydrogel under 808 nm NIR irradiation: the photothermal effect generated by the embedded MOF/LM nanocomposites rapidly elevates the local temperature, which further initiates the thermoresponsive volume shrinkage of the PNIPAM-based hydrogel network, forming the prerequisite for the coupling between wound contractile force and ion release. Digital photographs and corresponding SEM images (Fig. 3b) compare the macroscopic morphological changes of PGD and PGDML hydrogels with and without 808 nm NIR irradiation. For the PGD hydrogel without photothermal components, no obvious macroscopic or microscopic morphological changes were observed after NIR irradiation. In contrast, the PGDML hydrogel exhibited distinct volume shrinkage at the macroscopic level after NIR exposure (1.0 W cm^−2^, 5 min), and SEM characterization further revealed that the original interconnected porous network of the PGDML hydrogel underwent significant densification after NIR irradiation, providing direct morphological evidence for the light-induced contraction of the hydrogel matrix (Fig. 3b). Infrared thermal images (Fig. 3c) visualize the real-time spatiotemporal temperature evolution of the hydrogels under continuous NIR irradiation. The PGDML hydrogel showed a rapid, time-dependent and uniform temperature elevation, while the control PGD hydrogel exhibited negligible temperature change under identical irradiation conditions, demonstrating the excellent photothermal conversion capability of the MOF/LM nanocomposites immobilized in the hydrogel network.^38^ Moreover, we have measured the shrinkage ratio of the PGDML hydrogel under 808 nm NIR irradiation (1.0 W cm^−2^). Hydrogel discs (10 mm in diameter, 2 mm thick) were equilibrated in PBS (pH 7.4) at 25 °C for 24 h, then irradiated with NIR for different time. Photographs were taken and the diameter was measured. The PGDML hydrogel exhibited a time-dependent contraction, reaching 36.2% after 3 min and 54.8% after 5 min of NIR irradiation (Fig. S3). In contrast, the PGD hydrogel without MOF/LM nanoparticles showed negligible shrinkage (<3%) under identical conditions, confirming that the contraction is driven by the photothermal effect of the liquid metal core.

**Fig. 3:**
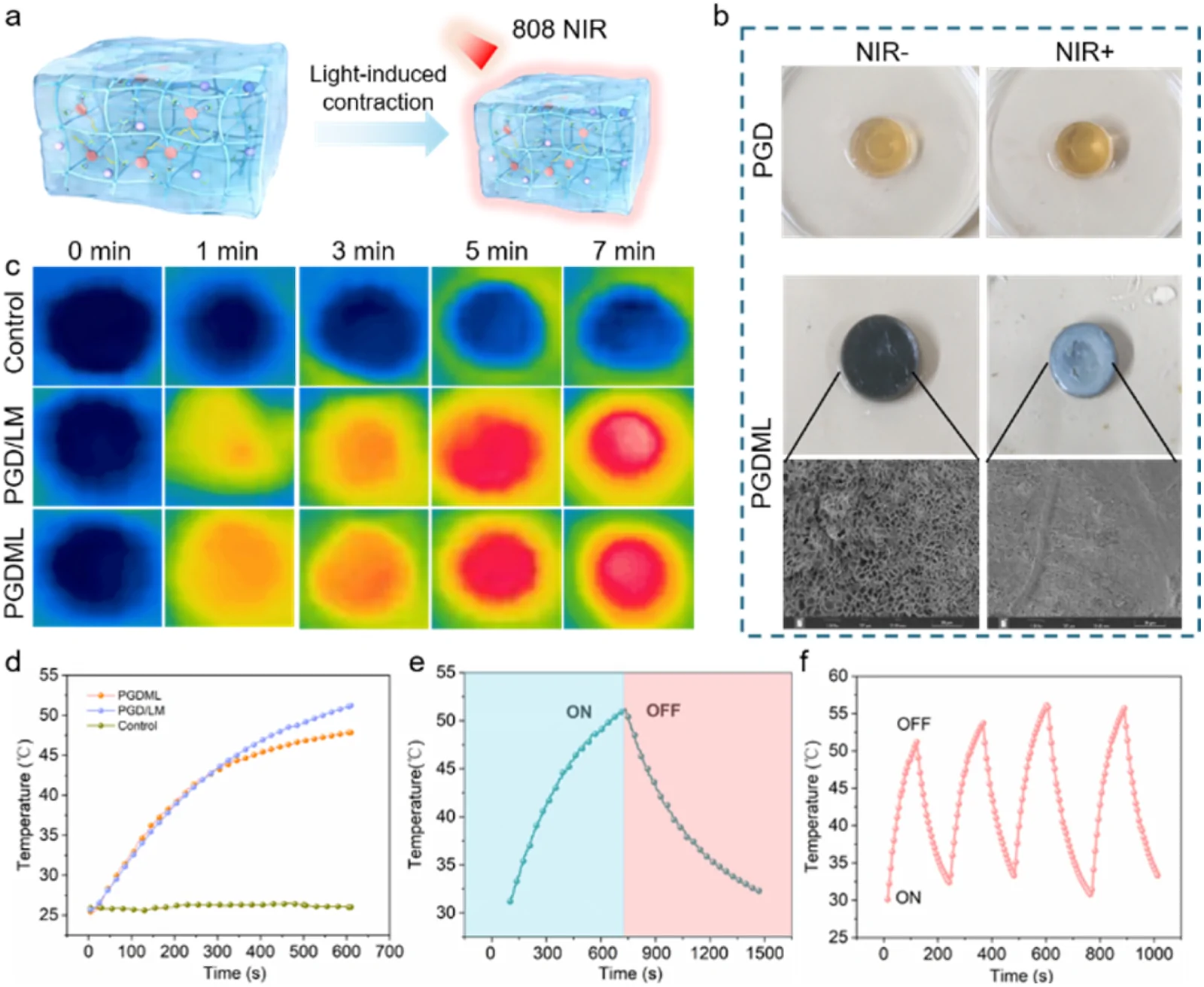
(a) Schematic illustration of PGDML hydrogel undergoing light-induced contraction under 808 nm NIR irradiation. (b) Digital photos of PGD and PGDML hydrogels before and after NIR irradiation. (c) Infrared thermal images of hydrogels at 0, 1, 3, 5 and 7 min upon NIR irradiation. (d) Temperature variation curves of different hydrogels with irradiation time under NIR laser. (e) Temperature on–off cycles of PGDML hydrogel with NIR laser switched on and off. (f) Multiple on–off cycles of temperature change of PGDML hydrogel.

Quantitative photothermal heating curves (Fig. 3d) further validated the superior photothermal performance of the PGDML hydrogel.^39^ Under NIR irradiation, the temperature of the PGDML hydrogel increased rapidly from 25 °C to over 50 °C within 600 s, while the pristine LM group showed a slightly lower heating rate, and the blank control group maintained a stable temperature near room temperature throughout the irradiation period. The reversible photothermal responsiveness of the PGDML hydrogel was evaluated *via* a single NIR laser on–off cycle test (Fig. 3e). When the NIR laser was switched on, the hydrogel temperature rapidly rose to ∼50 °C within 600 s; once the laser was turned off, the temperature cooled down to near the initial physiological temperature. Furthermore, multiple consecutive NIR on–off cycle tests (Fig. 3f) demonstrated the outstanding photothermal stability of the PGDML hydrogel. The hydrogel exhibited highly reproducible temperature elevation and cooling profiles over 5 repeated on–off cycles, with no obvious attenuation of the maximum temperature or response rate, verifying that the MOF/LM nanocomposites were stably anchored in the hydrogel network, and the hydrogel could maintain stable photothermal performance even after repeated NIR stimulation.

### 3.3 Tissue adhesive performance and ions release

The tissue adhesive performance of the composite hydrogel was characterized *via* lap shear assay (Fig. 4a and b).^40,41^ As shown in Fig. 4c, the PGDML hydrogel achieved the highest adhesion strength of ∼16 kPa on porcine skin, outperforming its adhesion on inorganic substrates including glass, aluminum, and wood. The robust adhesion is driven by abundant catechol groups from gelatin-DA in the hydrogel network, which form strong hydrogen bonds, covalent interactions, and electrostatic forces with amine and carboxyl groups on the tissue surface (Fig. 4d). Macroscopic adhesion images further verified that the hydrogel could form firm bonding with a wide range of substrates including glass, metal, plastic, rubber and porcine skin without detachment. Notably, the hydrogel maintained stable interfacial bonding on porcine skin under repeated dynamic deformations including bending, stretching and twisting, exhibiting excellent dynamic adhesion stability to meet the application requirements of highly mobile wound sites in clinical settings. Tensile tests revealed that the PGDML hydrogel exhibited improved tensile strength and stretchability compared with the PGD hydrogel (Fig. 4f), as the embedded MOF/LM nanocomposites act as physical crosslinkers to reinforce the hydrogel network.^42^ The hydrogel also showed excellent flexibility and fatigue resistance, withstanding repeated bending and stretching without structural damage (Fig. 4g), enabling it to adapt to skin deformation during physiological activity.

**Fig. 4:**
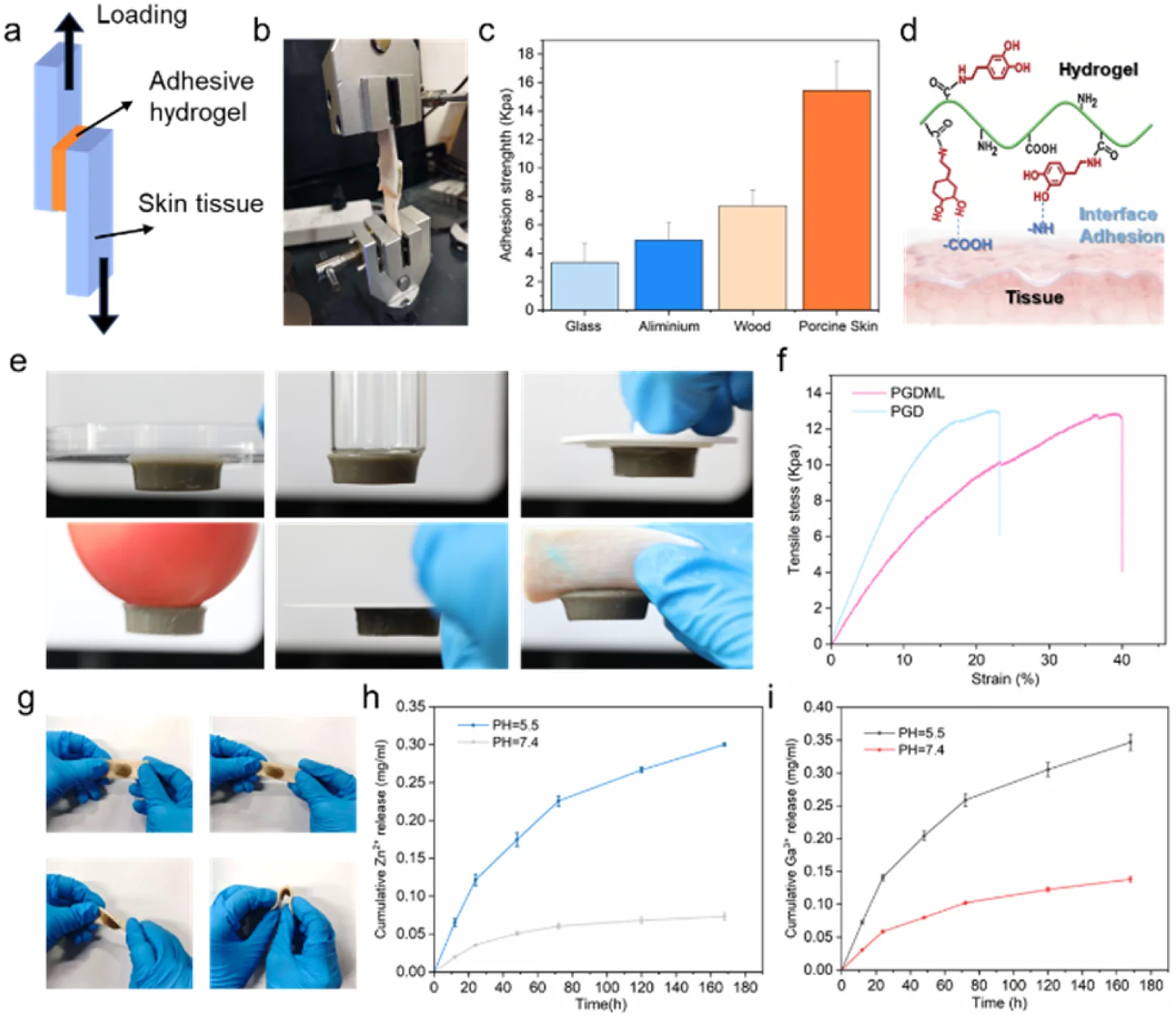
(a) Schematic diagram of the adhesion test between adhesive hydrogel and skin tissue. (b) Digital photo of the adhesion strength test setup. (c) Adhesion strength of the hydrogel on different substrates including glass, aluminum, wood and porcine skin. (d) Schematic illustration of the interfacial adhesion mechanism between hydrogel and tissue. (e) Photos showing the hydrogel adhering to various surfaces including glass, beaker wall, plastic sheet, rubber ball and ex vivo skin. (f) Tensile stress–strain curves of different hydrogel samples. (g) Photos demonstrating the hydrogel effectively adhering porcine skin and withstanding stretching, bending, and twisting. Cumulative release curves of Zn^2+^ (h) at and Ga^3+^ (i) at different pH (*n* = 3).

Furthermore, we investigated the pH-responsive ion release behavior of the PGDML hydrogel (Fig. 4h and i). Under acidic conditions (pH 5.5, mimicking the microenvironment of infected wounds), the cumulative release of Zn^2+^ and Ga^3+^ was significantly accelerated and enhanced compared with release under neutral physiological conditions (pH 7.4). This infection-responsive ion release is driven by accelerated degradation of the MOF framework in acidic media, enabling on-demand delivery of antibacterial ions at the infected site and minimizing non-specific side effects on normal tissues.^43^

### 3.4 *In Vitro* antibacterial and anti-biofilm activities

Bacterial infection and biofilm formation are the primary obstacles to clinical wound healing, which can lead to delayed wound closure, chronic inflammation, and even systemic sepsis. *E. coli* and *S. aureus* are the most common conditional pathogens in wound infections.^44^ In this study, the plate coating method and crystal violet staining method were used to systematically evaluate the bactericidal and anti-biofilm properties of the prepared hydrogels, with sterile PBS as the blank control group, pure PNIPAAm-gelatin-dopamine hydrogel as the PGD group, MOF/LM loaded composite hydrogel as the PGDML group, and PGDML hydrogel with NIR irradiation as the PGDML + NIR group.

As shown in the plate colony images and quantitative antibacterial rate results (Fig. 5a and b), the blank control group and pure PGD hydrogel group exhibited dense, numerous bacterial colonies of both *E. coli* and *S. aureus*, with an antibacterial rate of less than 10% for both strains. No statistically significant difference was observed between the PGD group and the control group, indicating that the pure PGD hydrogel has no clinically effective bactericidal activity. Although the catechol groups in dopamine have been reported to have weak bacteriostatic effects, this effect is too limited to achieve the bactericidal efficiency required for wound anti-infection, which is consistent with the performance of dopamine-modified hydrogels in previous relevant studies.

**Fig. 5:**
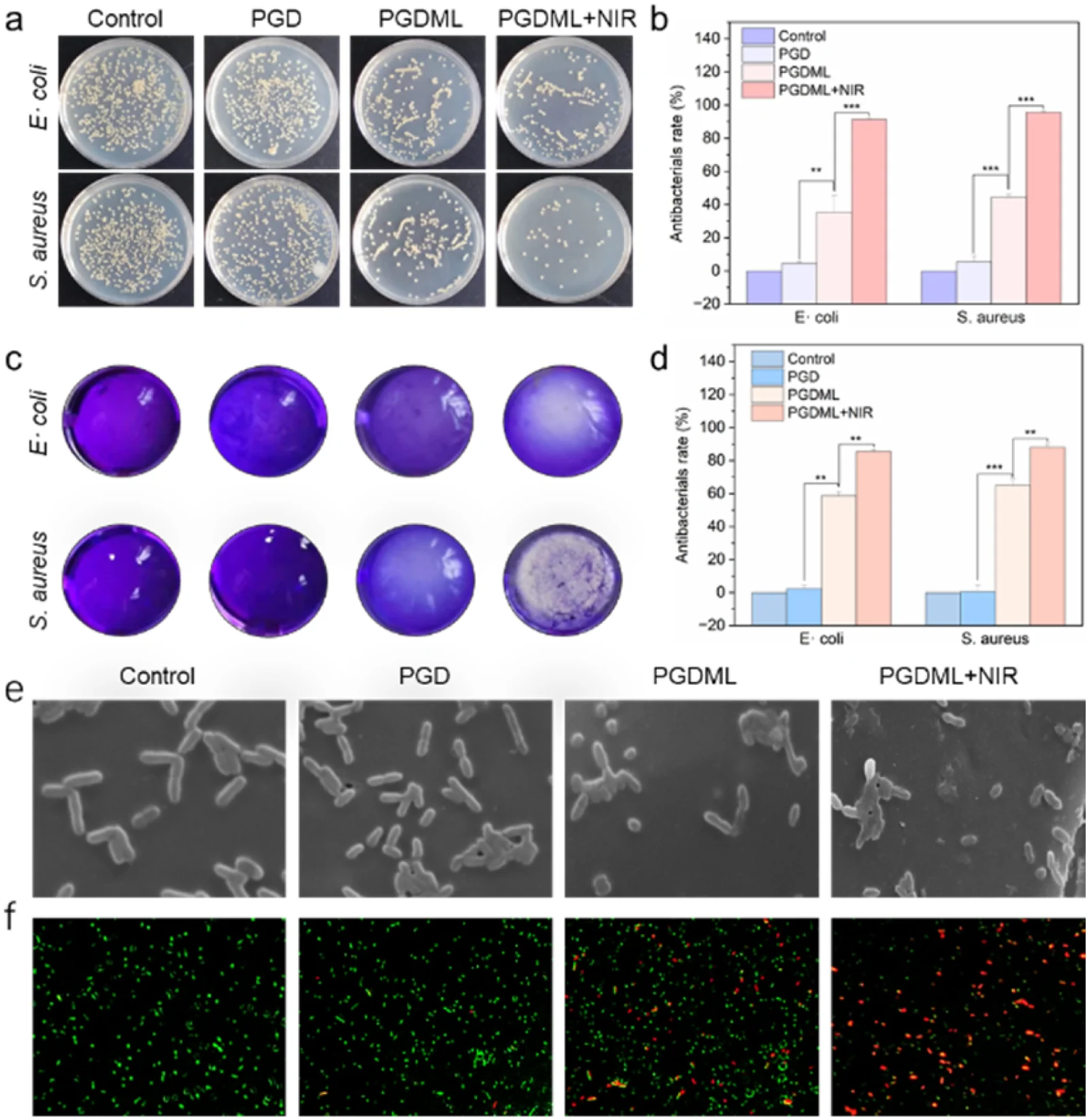
(a) Colony formation images of *E. coli* and *S. aureus* after co-cultured with control, PGD, PGDML, and PGDML under NIR irradiation. (b) Quantitative antibacterial rate histogram of different groups against *E. coli* and *S. aureus*. (c) live/dead bacterial staining fluorescence images of *E. coli* and *S. aureus* after treated with different formulations, live bacteria stained in blue. (d) Statistical histogram of the relative bacterial activity of different treatment groups towards *E. coli* and *S. aureus*. (e) *E. coli* morphology after different treatments. (f) Live/dead bacterial fluorescence staining.

In contrast, the PGDML group showed a significant reduction in colony number for both strains, with antibacterial rates of 35.2% against *E. coli* and 44.6% against *S. aureus*, respectively, which was extremely significantly higher than the PGD group. This intrinsic antibacterial activity was derived from the sustained release of active metal ions from MOF/LM nanoparticles uniformly dispersed in the hydrogel matrix. The ZIF-8 framework in the nanoparticles was gradually degraded in the aqueous physiological environment, continuously releasing Zn^2+^; meanwhile, the liquid metal core released Ga^3+^ in a sustained manner. Both Zn^2+^ and Ga^3+^ can directly disrupt the integrity of bacterial cell membranes, increase membrane permeability, cause the leakage of bacterial intracellular contents, and inhibit bacterial proliferation, thus exerting a stable and continuous bactericidal effect.

Notably, the PGDML + NIR group achieved nearly complete inactivation of bacteria, with antibacterial rates as high as 91.2% against *E. coli* and 95.7% against *S. aureus*, which was significantly superior to other groups. This excellent bactericidal performance came from the synergistic antibacterial effect of photothermal sterilization and metal ion chemical sterilization. On the one hand, the liquid metal core in the composite hydrogel has excellent photothermal conversion efficiency, and can rapidly generate local high temperature under NIR irradiation. The hyperthermia can directly cause irreversible denaturation of bacterial proteins and rupture of bacterial cell membranes, achieving rapid physical sterilization through the photothermal effect itself. On the other hand, the elevated temperature accelerates the degradation of the ZIF-8 framework and the ion release from the liquid metal core, leading to a rapid increase in the local concentration of Zn^2+^ and Ga^3+^ at the wound site, which further amplifies the chemical antibacterial effect of the metal ions. The dual effects of photothermal physical sterilization and ion chemical sterilization achieved a highly efficient synergistic antibacterial effect.

Compared with the inactivation of planktonic bacteria, the clearance of mature bacterial biofilms is more critical for the treatment of infected wounds.^45^ The dense extracellular polymeric substance (EPS) matrix of biofilms acts as a physical barrier to block the penetration of antibacterial substances, leading to high bacterial drug resistance and persistent wound infection. The crystal violet staining images and quantitative biofilm clearance rate results showed that the control group and PGD group presented uniform deep purple staining at the bottom of the wells, indicating that the pure PGD hydrogel had no obvious anti-biofilm activity (Fig. 5c and d). In comparison, the PGDML group showed significantly lighter purple staining, with biofilm clearance rates of 58.8% against *E. coli* and 65.3% against *S. aureus*, respectively, which was extremely significantly higher than the PGD group. This anti-biofilm effect was attributed to the sustained release of Zn^2+^ and Ga^3+^, which can inhibit the synthesis and secretion of EPS by bacteria, reduce the compactness of the biofilm structure, and kill the viable bacteria wrapped in the biofilm, thus effectively inhibiting the formation and maturation of bacterial biofilms. The PGDML + NIR group achieved highly efficient clearance of mature biofilms, with clearance rates of 85.4% against *E. coli* and 88.3% against *S. aureus*, respectively, which was extremely significantly higher than all other groups. This excellent anti-biofilm effect also came from the synergistic effect of photothermal and metal ions. The local hyperthermia generated by NIR irradiation can directly destroy the compact three-dimensional network structure of the biofilm, denature and degrade the EPS matrix, break the physical barrier that hinders the penetration of antibacterial substances, and enable Zn^2+^ and Ga^3+^ to rapidly penetrate into the deep layer of the biofilm. Meanwhile, the elevated temperature further accelerated the release of Zn^2+^ and Ga^3+^, forming a high-concentration ion microenvironment inside the biofilm, which thoroughly killed the persister bacteria embedded in the biofilm, and achieved efficient clearance of mature biofilms.

In addition, bacterial morphology was observed under SEM. As shown in Fig. 5e, untreated bacteria (control and PGD groups) exhibited typical intact morphology—*E. coli* presented smooth, rod-shaped surfaces with clearly and complete boundaries. In contrast, obvious collapse and structural disruption of bacterial membranes were observed in the PGDML group, and such membrane destruction was further aggravated upon NIR irradiation (PGDML + NIR). These observations provide direct visual evidence that the synergistic effect of photothermal heating and accelerated ion release leads to catastrophic loss of membrane integrity.

Consistently, live/dead bacterial fluorescence staining relies on membrane permeability to differentiate live and dead bacteria. Consistently, live/dead staining results demonstrated dominant green fluorescence (live bacteria) in control and PGD groups; the proportion of red fluorescence (dead bacteria with compromised membrane) increased in the PGDML group, and nearly all bacteria exhibited red signals in the PGDML + NIR group. These results collectively confirm that the ion-mechanical coupling effect triggered by NIR stimulation can severely disrupt bacterial membrane integrity, providing direct experimental evidence supporting our proposed antibacterial mechanism.

### 3.5 *In Vivo* antibacterial efficacy and wound healing assessment

Bacterial infection is a major clinical challenge in cutaneous wound repair, which triggers persistent inflammatory responses, disrupts the ordered wound healing process, and leads to delayed wound closure, chronic non-healing ulcers, and even life-threatening systemic sepsis. We established a mouse full-thickness skin defect wound infection model to systematically evaluate the *in vivo* antibacterial efficacy and wound healing promotion effect of the prepared hydrogels (Fig. 6a).^46^ Infrared thermal imaging results (Fig. 6b) verified the reliable *in vivo* photothermal conversion performance of the PGDML hydrogel. Under NIR irradiation, the PGDML hydrogel at the wound site achieved a rapid temperature rise within 5 min, and maintained a stable local hyperthermia during the whole irradiation period. This result is highly consistent with our *in vitro* photothermal test results, confirming that the PGDML hydrogel has excellent and reliable photothermal conversion efficiency even in the complex *in vivo* wound environment. Macroscopic wound appearance results (Fig. 6c) intuitively demonstrated the excellent wound healing promotion effect of the PGDML + NIR hydrogel. On day 10 after treatment, the wound in the PGDML + NIR group was nearly completely closed with full re-epithelialization, while the control group and PGD group still had large unhealed wound areas accompanied by obvious redness. On day 14, the PGDML + NIR group achieved complete wound healing with minimal scar formation (Fig. 6d). Quantitative analysis of wound closure rate reveals a time-dependent increase in wound healing for all groups over the 14-day treatment period, with the PGDML + NIR group displaying significantly accelerated wound closure and the highest healing efficacy (Fig. S4). This superior pro-healing effect comes from the combined advantages of the composite hydrogel: efficient antibacterial effect eliminates the primary cause of delayed healing, terminates persistent inflammatory stimulation, and enables the wound to transition to the proliferation phase in time. The wound area at various time points further confirmed the positive effect of the PGDML hydrogel on the healing of infectious chronic wounds.

**Fig. 6:**
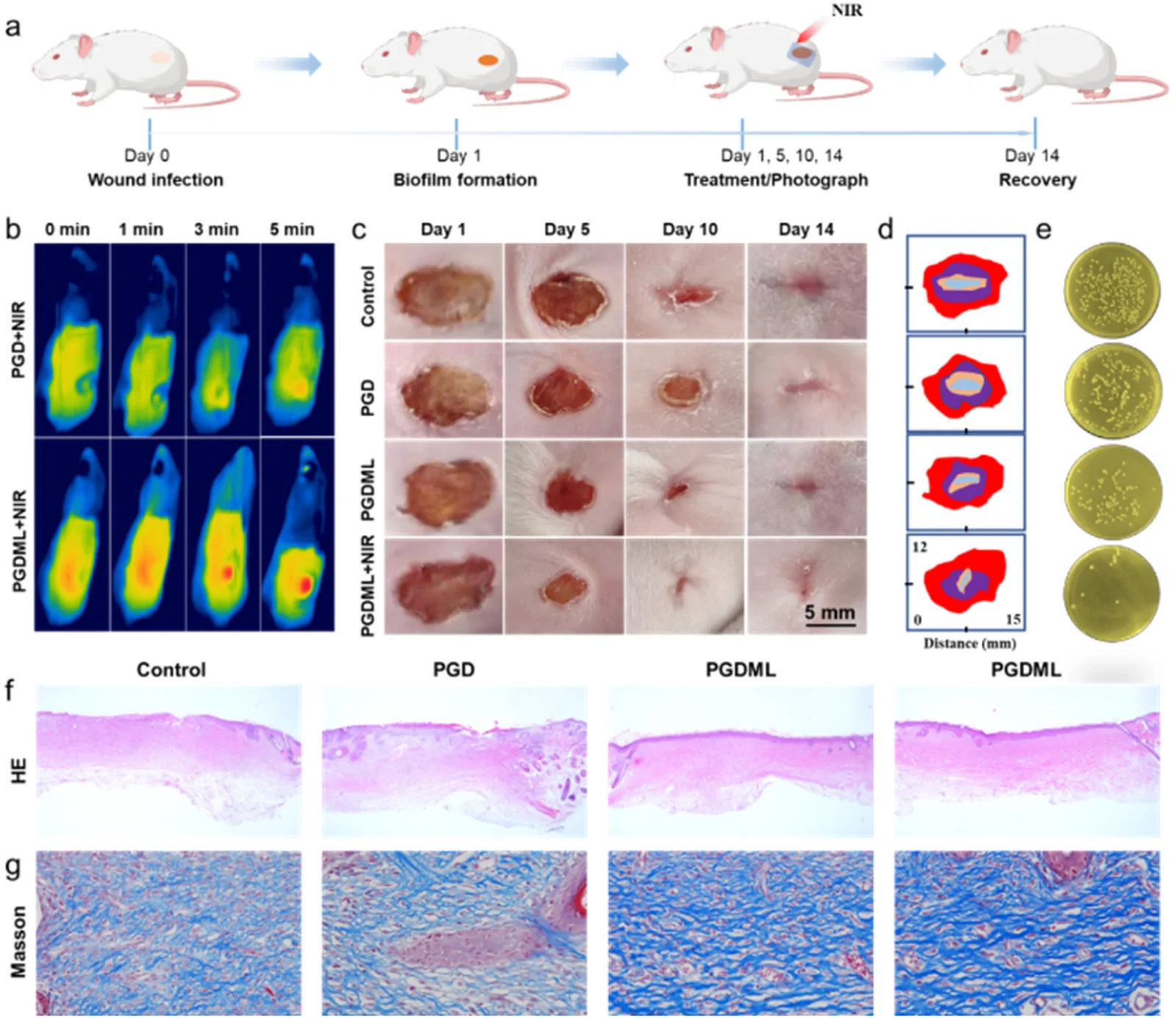
(a) Schematic illustration of the *in vivo* wound infection treatment process: mice were infected with bacteria on day 0, biofilms formed on day 1, and treated with NIR irradiation on days 1, 5, 10, 14, and recovered on day 14. (b) Infrared thermal images of mice under NIR irradiation at different time points. (c) Wound healing status of mice in Control, PGD, PGDML and PGDML + NIR groups on days 1, 5, 10 and 14. (d) Quantitative analysis of wound area reduction in each group during treatment. (e) Bacterial colony culture results from wound secretions of each group after treatment. (f) H&E staining images of wound tissue sections in each group after healing. (g) Masson staining images of wound tissue sections, showing collagen deposition and tissue remodeling in each group.

The *in vivo* antibacterial effect was further confirmed by wound tissue colony counting assay (Fig. 6e). On day 5 after treatment, a large number of dense bacterial colonies were observed in the tissue homogenate plates in the control group and pure PGD hydrogel group, indicating persistent severe bacterial infection in the wounds, and the pure PGD hydrogel had no effective *in vivo* antibacterial ability. In contrast, the PGDML group showed a significant reduction in bacterial load, proving the intrinsic antibacterial activity of MOF/LM nanoparticles *via* sustained release of active metal ions. Impressively, the PGDML + NIR group achieved an *in vivo* antibacterial rate of over 90%, with almost no residual bacteria detected at the wound site, which was significantly superior to all other groups. This excellent antibacterial effect is attributed to the synergistic mechanism: NIR-induced local hyperthermia directly causes bacterial protein denaturation and membrane rupture to achieve physical sterilization, and simultaneously accelerates the degradation of the ZIF-8 framework and the release of Zn^2+^ and Ga^3+^, amplifying the chemical bactericidal effect of metal ions. This antibiotic-free antibacterial strategy effectively avoids the risk of bacterial drug resistance caused by long-term antibiotic use, which has significant advantages for clinical anti-infection treatment.

To directly demonstrate that mechanical contraction and ion release act synergistically rather than as two parallel independent processes, we performed an additional control experiment in which a flexible heating pad was placed on top of the PGD hydrogel (without MOF/LM) to provide purely thermal stimulation. This heating pad was calibrated to match the temperature profile and shrinkage ratio of the PGDML + NIR group, thereby isolating the contribution of mechanical contraction alone while eliminating any ion release.

As shown in Fig. S5 (representative wound images and the closure rate), the PGDML + NIR group exhibited the fastest wound closure, with nearly complete re-epithelialization by day 10 and minimal scar formation by day 14. The “Contraction Only” group showed moderate acceleration of healing compared with the control group, confirming that mechanical contraction alone provides a beneficial effect. However, its healing rate was significantly slower than that of the PGDML + NIR group. Importantly, the therapeutic effect of PGDML + NIR exceeded the simple additive effect of contraction alone and ion release alone, confirming a true synergistic interaction. These results provide direct evidence that mechanical contraction and ion release are functionally coupled, not independent.

The cytocompatibility of the PGDML hydrogel was evaluated using L929 mouse fibroblasts *via* MTT assay. Cells were incubated with hydrogel extracts at concentrations ranging from 1% to 100% for 24 h. As shown in Fig. S6, cell viability remained above 80% in all treatment groups, even at the highest concentration (100%), with no statistically significant difference compared with the control group, confirming that the PGDML hydrogel is safe for wound-healing applications. Histological analysis further confirmed the high-quality tissue regeneration effect of the PGDML + NIR hydrogel. Hematoxylin–eosin (H&E) staining results (Fig. 6f) showed that on day 14, the PGDML + NIR group formed a complete and well-structured stratified squamous epithelium. In contrast, the control and PGD groups had incomplete epidermal regeneration, inflammatory cell infiltration, and no obvious skin appendage regeneration. Masson staining (Fig. 6g) revealed that the PGDML + NIR group had dense and regularly arranged collagen fibers with high content, which was consistent with the collagen structure of normal skin, while the control and PGD groups showed sparse and disordered collagen deposition, a typical feature of immature scar tissue.

To evaluate the systemic biosafety of the PGDML hydrogel, major organs (heart, liver, spleen, lung, and kidney) were harvested after 14 days of treatment and subjected to H&E staining. As shown in Fig. S7, no obvious histopathological abnormalities, tissue damage, or inflammatory cell infiltration were observed in the PGDML + NIR group compared with the control group. In summary, the PGDML composite hydrogel combined with NIR irradiation can achieve efficient *in vivo* antibacterial effect, eliminate wound infection, accelerate wound closure, and promote high-quality skin tissue regeneration with functional recovery. This multifunctional hydrogel integrates multiple core functions required for clinical wound infection treatment, and has great clinical transformation potential for the management of bacteria-infected acute and chronic wounds.

## 4. Conclusion

In conclusion, we report a NIR-responsive adhesive hydrogel (PGDML) featuring a core mechanism of coupling between antibacterial ion release and wound contractile force for efficient bacteria-infected wound repair. This hydrogel, fabricated by integrating MOF-caged liquid metal nanocomposites into a gelatin-PNIPAM dual network, exhibits robust tissue adhesion and precise NIR responsiveness. Upon NIR irradiation, photothermal heating simultaneously drives hydrogel contraction to generate wound contractile force and accelerates bactericidal ion release; this ion force coupling effect achieves synergistic high-efficiency bacterial eradication, and markedly accelerates re-epithelialization, collagen deposition and full-thickness infected wound healing *in vivo*. This work presents a generalizable ionic force coupling strategy for engineering smart responsive biomaterials, addressing a critical unmet need in the management of drug-resistant bacterial infections and chronic wound repair, with broad application potential for wound regenerative medicine.

## Author contributions

Xiaoxiao Hu: writing – original draft, validation, funding acquisition, data curation, software. Lemin Zhang: writing – review & editing, visualization, project administration, methodology, conceptualization. Ruixin Hao: visualization, methodology, investigation. Zeyu Wang: software, investigation. Qiuhong Zhang: methodology, formal analysis. Zhicheng Gao: supervision, methodology. Wei Yang: supervision, project administration, conceptualization. Yongping Ma: supervision, project administration, writing – review & editing, validation, funding acquisition.

## Conflicts of interest

The authors declare that they have no known competing financial interests or personal relationships that could have appeared to influence the work reported in this paper.

## Supplementary Material

RA-016-D6RA07136H-s001

## Data Availability

Data will be made available on request. Supplementary information (SI) is available. See DOI: https://doi.org/10.1039/d6ra07136h.
