# Supplementary material for: NIR-triggered gelatin-based hydrogel for ion-mechanical synergistic antibacterial therapy and infected wound closure
Source: RSC Adv. 2026 Oct 19;16(48):64327–37. doi: 10.1039/d6ra07136h (PMC13678685; doi:10.1039/d6ra07136h)
Supplement: Supplementary file 1 [file RA-016-D6RA07136H-s001.pdf]

Supplementary Information (SI) for RSC Advances.
This journal is © The Royal Society of Chemistry

Supporting Information

NIR-triggered gelatin-based hydrogel for ion-mechanical synergistic antibacterial therapy and infected wound closure

Xiaoxiao Hu[a], Lemin Zhang[b,*], Ruixin Hao[c], Zeyu Wang[d], Qiuhong Zhang[a], Zhicheng Gao[a], Wei Yang[e,*], Yongping Ma[d,f,*]

[a] *Department of Clinical Pharmacy, The No.2 Hospital of Baoding, Baoding 071052, People's Republic of China*

[b] *School of Mechanical Engineering, Dalian University of Technology, Dalian 116024, China*

[c] *School of Physics and Materials, Nanchang University, Nanchang 330031, China*

[d] *College of Stomatology, North China University of Science and Technology, Tangshan 063200, People's Republic of China*

[e] *Respiratory Medicine, The No.2 Hospital of Baoding, Baoding 071052, People's Republic of China*

[f] *Department of Stomatology, The No.2 Hospital of Baoding, Baoding 071052, People's Republic of China*

* Corresponding authors.

E-mail address: zlmhdjc@mail.dlut.edu.cn (L. Zhang), hdfyyw@sina.com (W. Yang), 3201165077@qq.com (Y. Ma).

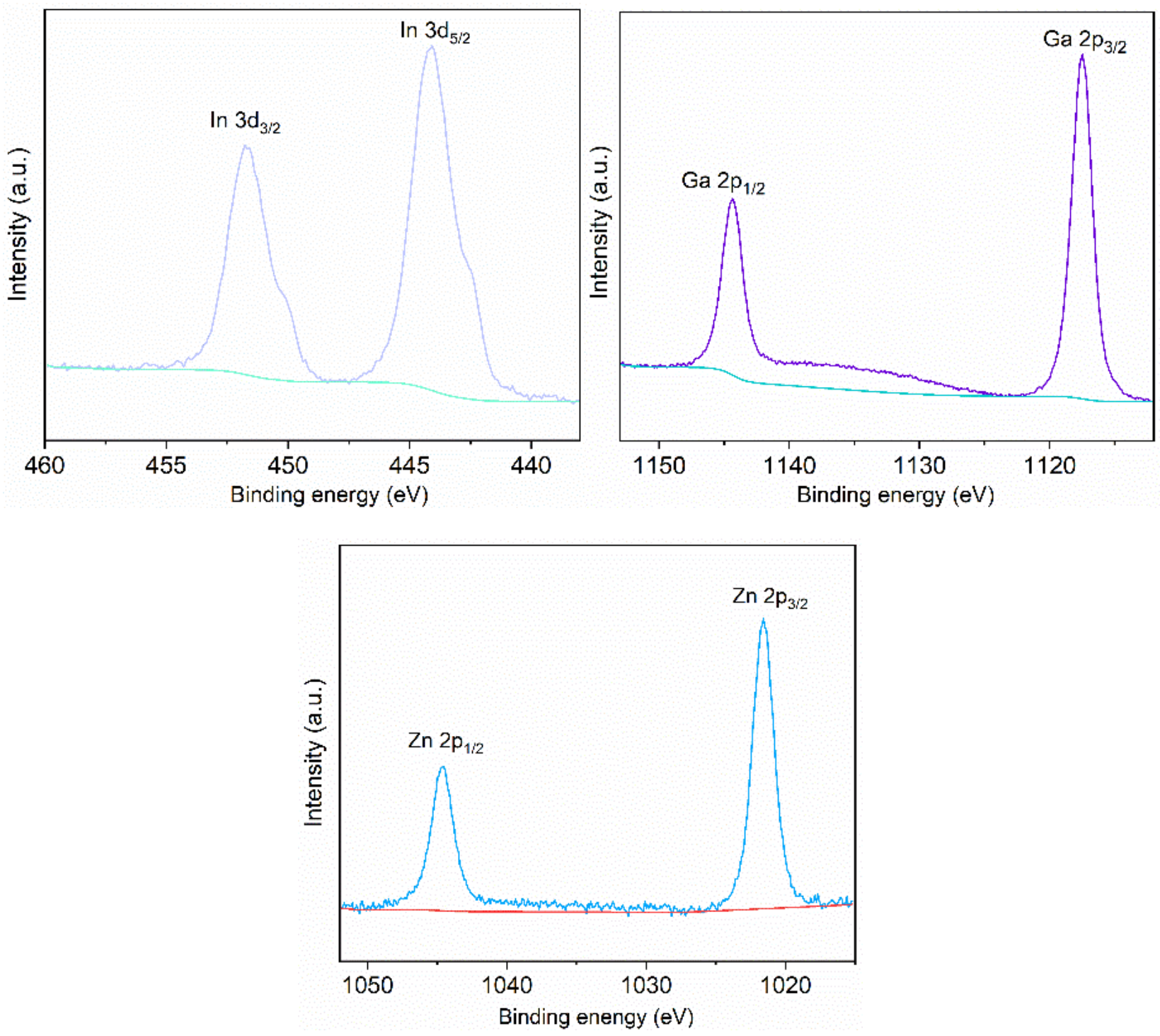

Fig. S1 High-resolution XPS spectra of MOF/LM nanocomposites: In 3d (top left), Ga 2p (top right), and Zn 2p (bottom), verifying the successful integration of LM and MOF functional components.

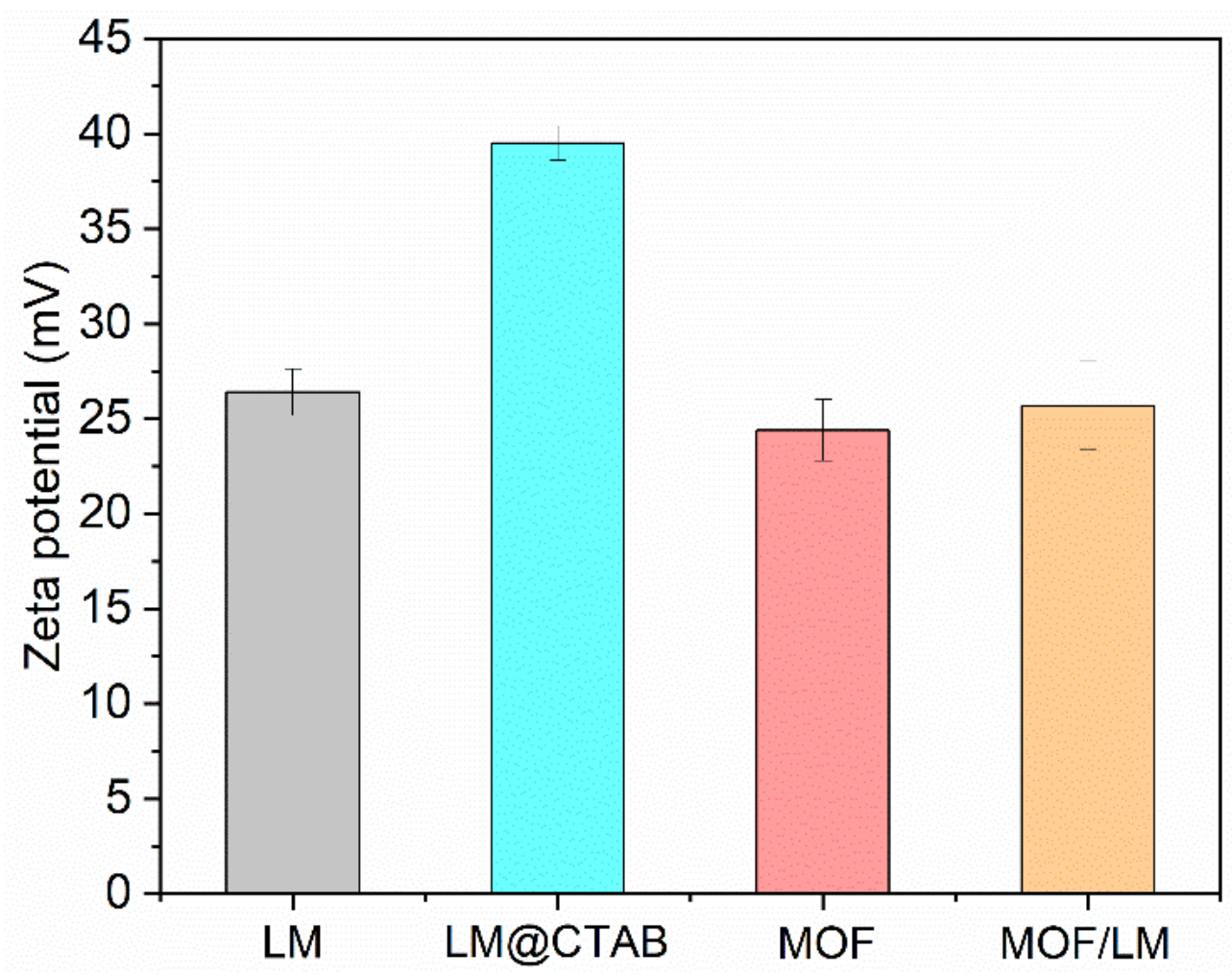

Fig. S2 Zeta potential of the as-prepared nanomaterials in aqueous dispersion, including pristine liquid metal (LM), CTAB-functionalized liquid metal (LM@CTAB), pure metal-organic framework (MOF) nanoparticles, and core-shell MOF/LM nanocomposites.

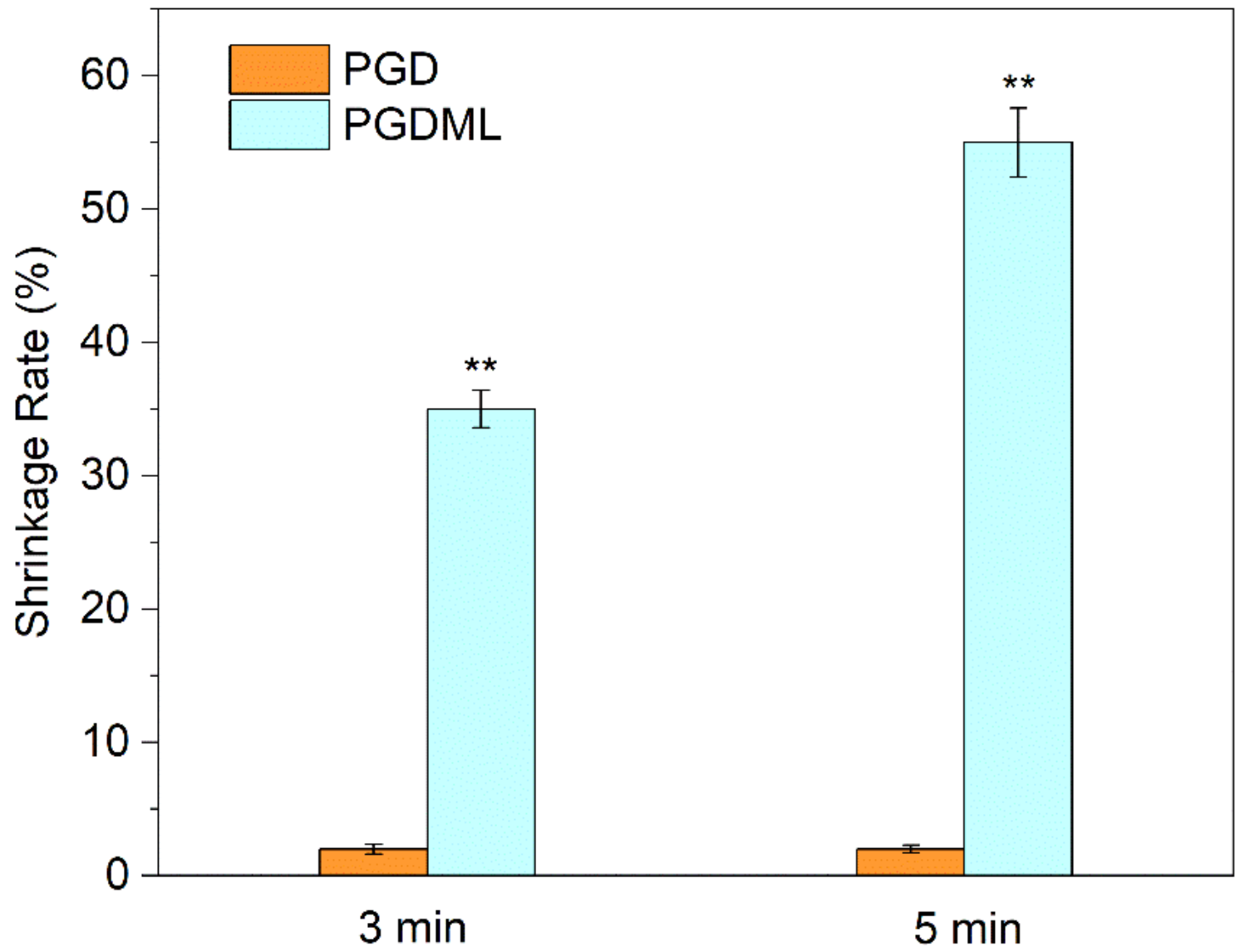

Fig. S3 Shrinkage rates of PGD and PGDML hydrogels after 808 nm NIR irradiation (1.0 W·cm^{-2}).

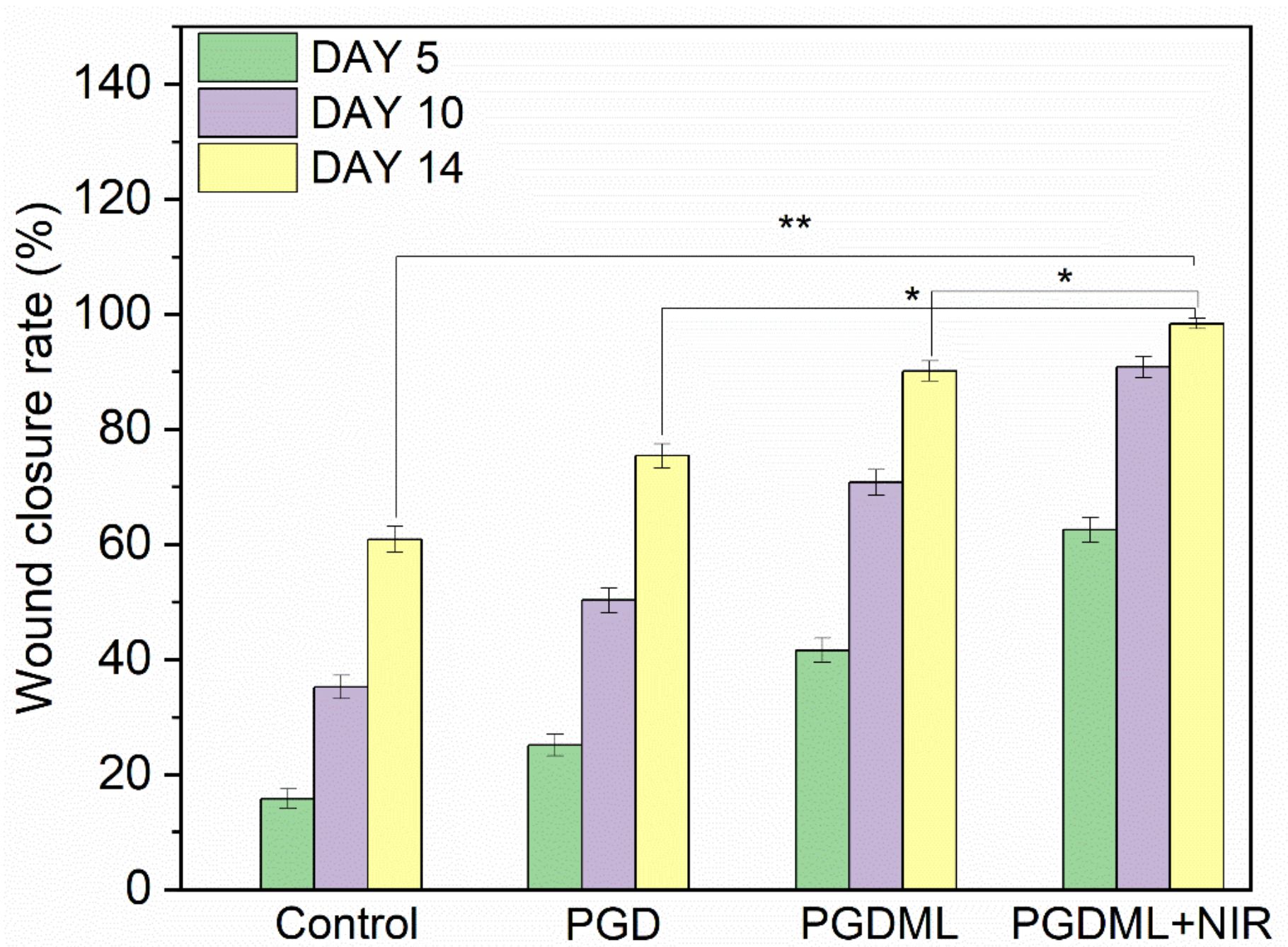

Fig. S4 Wound closure rate of infected wounds in the Control, PGD, PGDML, and PGDML+NIR groups at day 5, 10 and 14.

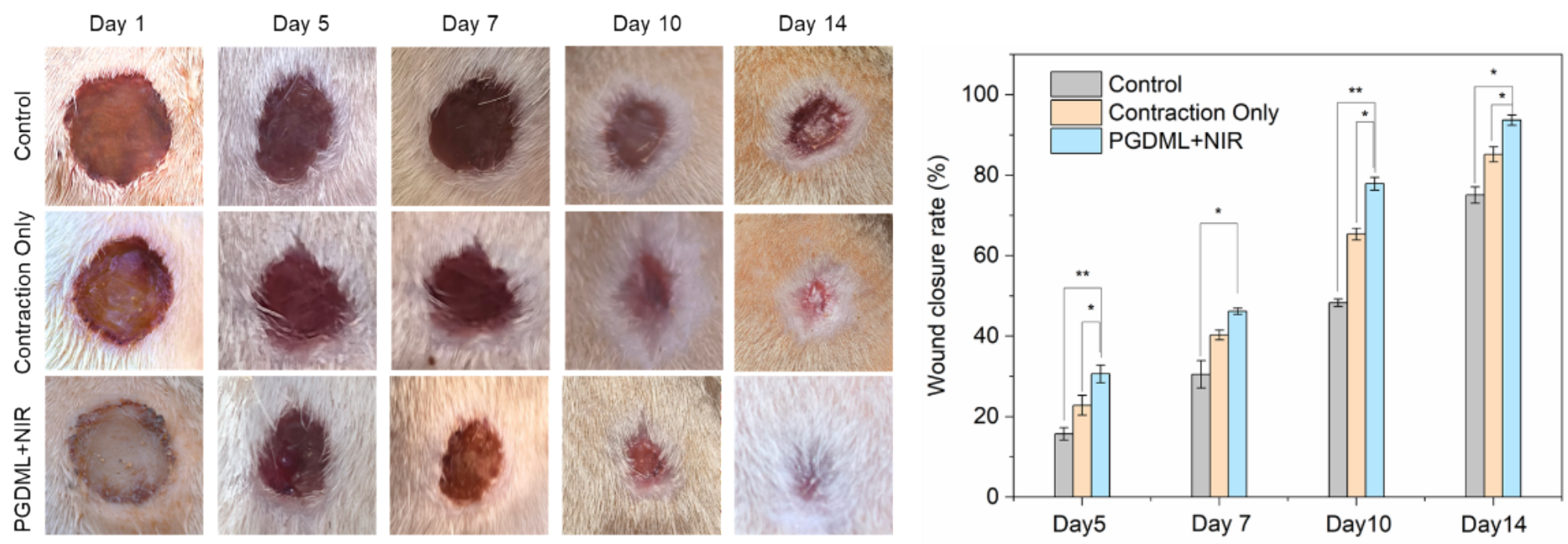

Fig. S5 (a) Representative photographs of MRSA-infected wounds treated with Control, Contraction Only (PGD hydrogel with a heating pad), and PGDML+NIR on days 1, 5, 7, 10, and 14. (b) Quantitative analysis of wound closure rates at days 5, 7, 10, and 14. Data are presented as mean ± SD ($n = 5$ per group).

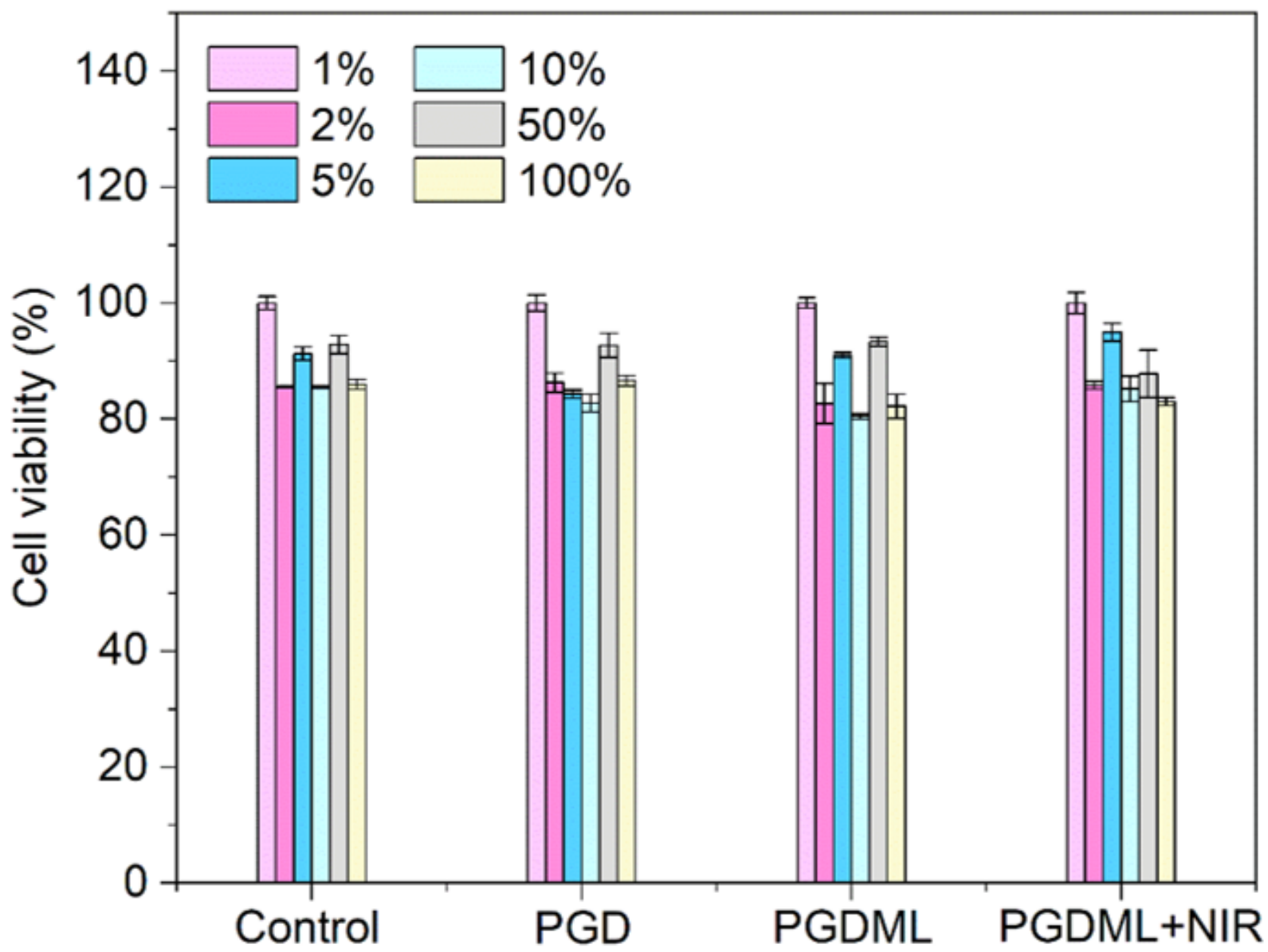

Fig. S6 Relative viability of primary rat dermal fibroblasts (L929).

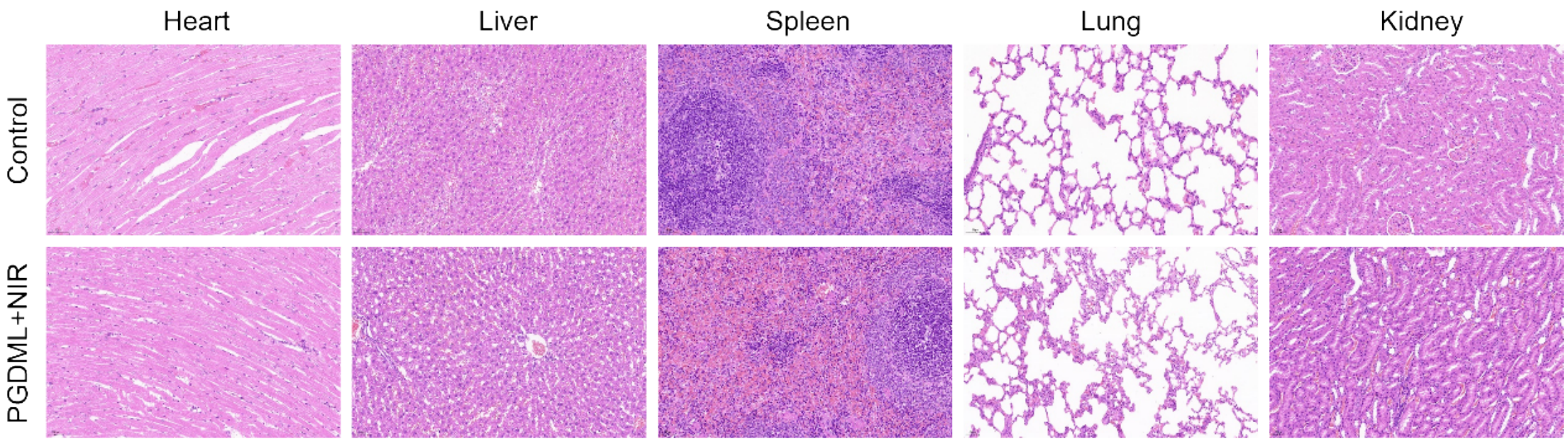

Fig. S7 Representative hematoxylin and eosin (H&E) staining images of major organs (heart, liver, spleen, lung, and kidney).
